# A school-based nutrition education program involving children and their guardians in Japan: facilitation of guardian-child communication and reduction of nutrition knowledge disparity

**DOI:** 10.1186/s12937-021-00751-z

**Published:** 2021-11-18

**Authors:** Keiko Asakura, Sachie Mori, Satoshi Sasaki, Yuji Nishiwaki

**Affiliations:** 1 Department of Environmental and Occupational Health, School of Medicine, Toho University, 5-21-16, Omori-nishi, Ota-ku, Tokyo, 143-8540 Japan; 2 Department of Social and Preventive Epidemiology, School of Public Health, the University of Tokyo, 7-3-1, Hongo, Bunkyo-ku, Tokyo, 113-0033 Japan

**Keywords:** Nutrition education, Primary school, Family communication, Disparity reduction

## Abstract

**Background:**

Since the risk of noncommunicable diseases is closely associated with dietary intake, it is important to establish healthy dietary habits in childhood. Although several dietary education programs for children have been attempted, their implementation at school was often difficult due to overcrowded study curricula. We developed a new program which included homework for children and guardians, and evaluated its effect. Determinants of the effect were also investigated.

**Methods:**

The school-based nutrition education program including a 45-min lecture, a series of homework assignments involving children and guardians, and two handouts was implemented in 14 public primary schools in Japan. Seven schools each underwent the intervention in an alternating manner. Nutrition knowledge (percentage (%) of correct answers in the nutrition knowledge questionnaire) and attitude/behavior toward diet was evaluated three times (May (baseline), October, February) as outcomes. These factors and their changes following the intervention were assessed by linear mixed models to adjust for individual factors, with consideration to clustering of the participants and repeated measurements.

**Results:**

In total, 2227 children aged 10–12 years and their guardians participated. All schools completed the program. Children’s nutrition knowledge level was significantly increased (8.7%, 95% confidence interval [7.7–9.7]) following the intervention. Communication between children and their guardians, which was positively related with nutrition knowledge, was facilitated by the intervention. The increase in nutrition knowledge was greater among children with a lower knowledge level at baseline.

**Conclusions:**

This school-based nutrition education program was effective and feasible. Appropriate teaching materials for homework can reduce the burden on schools and facilitate communication between children and guardians. Public schools can be crucial venues for decreasing disparities in nutrition knowledge.

**Trial registration:**

This study was registered as an intervention study in the UMIN Clinical Trials Registry (trial ID: UMIN000029252) on Sep 22, 2017.

**Supplementary Information:**

The online version contains supplementary material available at 10.1186/s12937-021-00751-z.

## Introduction

Recognizing the close association of dietary habits with risk of noncommunicable diseases [1], several dietary education programs to improve diet quality have been established and evaluated, particularly in children. While reviews [2, 3] report that most interventions were effective to some extent - such as in increasing physical activity, reducing obesity, increasing vegetable consumption, and increasing nutrition knowledge - the best method is still under debate. Some groups reported that their nutrition education program affected both children’s nutrition knowledge and dietary behavior, such as vegetable intake [4–6]. On the other hand, it is known that population approaches to tackling public health issues sometimes exacerbate health disparities, because this kind of intervention more easily reaches people who are health-conscious and have a better socioeconomic status [7].

We and others have speculated that this disparity might be decreased by implementing nutrition education in public schools, given that most children (98.8% of primary school children in 2018 [8]) are enrolled and can be educated in the same way. The study curriculum at school is already overcrowded, however, and any attempt to establish an efficient nutrition education program must be done with minimum burden on schools [2]. Recently, we developed a new nutrition education program for Japanese primary school children which teaches about nutrition and its health effects to prevent future noncommunicable diseases, such as hypertension or cardiovascular disease, with nutrition teachers working in a prefecture in the Kanto area (central part of the main island), Japan. The program includes a lecture at school, and also homework to minimize teaching time at school. The children were asked to do the homework with their guardians, both to facilitate communication between them at home and to indirectly increase the guardians’ nutrition knowledge. Involvement of guardians in such programs is thought to be important [9], because they usually prepare meals for their children and eat together with them.

The nutrition teachers and our research team cooperated to implement the program in public primary schools. In this intervention study, we quantitatively evaluated whether the program increased children’s nutrition knowledge using nutrition knowledge questionnaires previously developed for Japanese primary school children [10]. We also investigated the change in attitude/behavior toward diet and factors affecting the effect of the education program.

## Methods

### Participants and study outline

**Fig. 1:**
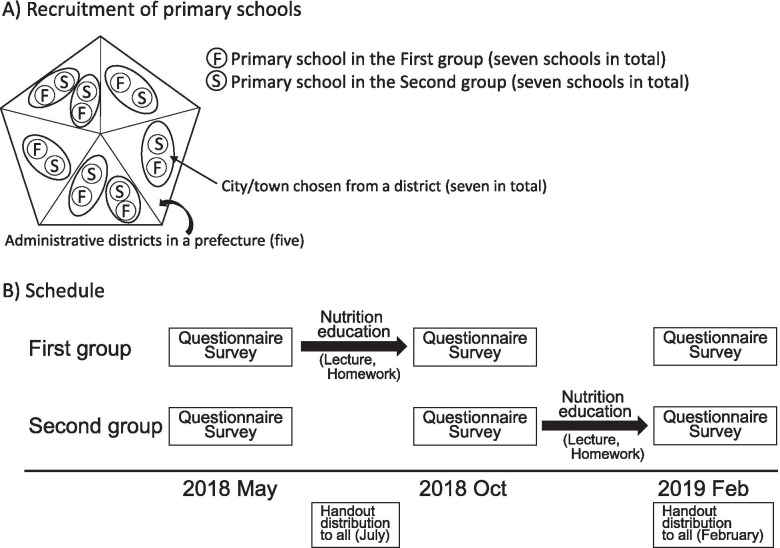
Outline of the study. **A** Recruitment of primary schools. Seven cities and towns were chosen from each of five administrative districts in the prefecture. Two public primary schools with similar characteristics were then selected from each city/town. For the two schools from each city/town, one was assigned to the First group and the second to the Second group, such that the First and Second groups included seven schools each. **B** Schedule. Questionnaire surveys were performed three times (May, October, and February). The nutrition education program (intervention) was given between the surveys. The intervention was implemented between June and September for the First group and between October and January for the Second group in an alternating manner

The study was implemented in a prefecture in the Kanto area, the central part of the main island of Japan. Seven cities and towns were chosen from each of five administrative districts in the prefecture, based on the feasibility of the survey (Fig. 1-A). Two public primary schools with similar characteristics (e.g. number of enrolled children, location (urban/rural)) were then selected from each city/town by the municipal boards of education. These 14 primary schools enrolled 2650 children as 5th and 6th graders (10–12 y/o) in April, 2018, all of whom were recruited into the study. At the same time, all of their guardians, most of whom were the main preparers of meals for the children, were recruited. No exclusion criteria were set, because the study included a nutrition education program at school and all children need to be educated equally in public schools. This study was conducted in accordance with the guidelines laid down in the Declaration of Helsinki and all procedures involving study participants were approved by the Ethics Committee of the Faculty of Medicine, Toho University (approval of revised version: no. A19003_A17043 on Apr.17, 2019; first approval: no. A17043 on Sep 6, 2017). The study was registered as an intervention study in the UMIN Clinical Trials Registry (trial ID: UMIN000029252) on Sep 22, 2017. Written informed consent for the children was obtained from their guardians. Regarding the guardians themselves, completion and return of the questionnaires was not mandatory, and those who did so were deemed to have consented to participation after detailed explanation about the study.

Surveys were performed three times (May, October, and February (Fig. 1-B)). The nutrition education program (intervention) was given between the surveys. For the two schools from each city/town, one was assigned to the First group and the second to the Second group as convenient with the schools’ schedules, such that the First and Second groups included seven schools each. The intervention was implemented between June and September for the First group and between October and January for the Second group.

### Questionnaires

In each survey, two questionnaires were distributed, one for dietary assessment and the second to measure nutrition knowledge and enquire about basic characteristics and attitudes/behaviors toward diet. Intake of nutrients and foods by the guardians and children was assessed using a brief-type, self-administered diet history questionnaire (BDHQ for adults and BDHQ15y for school children and adolescents). The BDHQs have been described in detail elsewhere [11–13]. Briefly, they are four-page fixed-portion questionnaires that ask about the consumption frequency of selected foods commonly consumed in Japan, general dietary behavior, and usual cooking methods to estimate the dietary intake of foods and beverage items during the preceding month. Estimates of daily intake for foods, energy, and selected nutrients were calculated using an ad hoc computer algorithm for the BDHQ and BDHQ15y based on the Standard Tables of Food Composition in Japan [14]. Both questionnaires have been appropriately validated [11–13], and used in several epidemiologic studies [15, 16]. The children were asked to answer the BDHQ15y with their guardians at home. The reporting sheet for intake was individually returned to the children and the guardians 1 month after the survey and used in subsequent nutrition education classes at the school.

The nutrition knowledge questionnaires have been described in detail elsewhere [10]. We used the questionnaire for higher graders (grade 4 to 6) with the children, and that for adults with the guardians. In brief, these included the following sections: 1) knowledge about foods as nutrient sources; 2) physiological functions of nutrients in the body; 3) awareness of dietary recommendations (only for adults); and 4) relationship between nutrients and health outcomes. We calculated the percentage (%) of correct answers (regarded as nutrition knowledge level) in the questionnaires. In total, 27 items were included in the questionnaire for the children, and divided into 51 yes/no questions to calculate the percentage of correct answers. In the questionnaire for the guardians, 84 items (yes/no questions) were included and used to calculate the percentage of correct answers.

Further, information about attitude and behavior toward diet was also collected using the children’s and guardians’ responses to whether they agreed (yes) or disagreed (no) with the following four statements: 1) Being careful about what you eat is beneficial for you; 2) Being careful about what you eat earns you esteem from those around you; 3) Being careful about what you eat places a burden on you; and 4) You discuss meals, food, nutrition, etc. with your guardians (or ‘your child’ in the statements for the guardians). These four questions were asked three times (in May, October, and February). The guardians answered two additional statements in May: 5) My current dietary habits are firmly fixed, so changing them is difficult; and 6) My dietary habits are healthy, so there’s no need to change them. In addition, the guardians were also asked about subjective socioeconomic status (SES), with answers selected from the five choices of very straitened, straitened, average, affluent, very affluent.

### Intervention

The nutrition education program consisted of a 45-min lecture at school, a series of teaching materials for homework, and two handouts. The lecture focused on the relationship between nutrient intake and lifestyle-related diseases and was formally given in the classroom by the homeroom teacher or a nutrition teacher. Summarized results of the dietary assessment (intake of protein, fat, carbohydrate, dietary fiber, and sodium) among the children in each class were presented, and every child compared the summaries with his/her own intake. Each student then chose a goal to help them practice better dietary habits, and kept a one-week short diary to review their diet. The teaching materials for homework included six topics: classification of major nutrients, carbohydrate, fat, calcium, salt, and vitamins. Basically, the materials were distributed weekly or biweekly. The children brought the materials home, read them, and answered some quizzes. These homework and quizzes covered the same topics as the nutrition knowledge questionnaire, but did not include the same questions. The guardians then checked the materials with their children and signed them to indicate approval. The children then submitted the materials to the school, where quizzes for review was again conducted. The handouts mainly targeting the guardians were distributed in July and February irrespective of the timing of the intervention. These handouts summarized the results of the dietary assessment and provided information about the prevention of lifestyle-related diseases.

### Statistical analysis

**Fig. 2:**
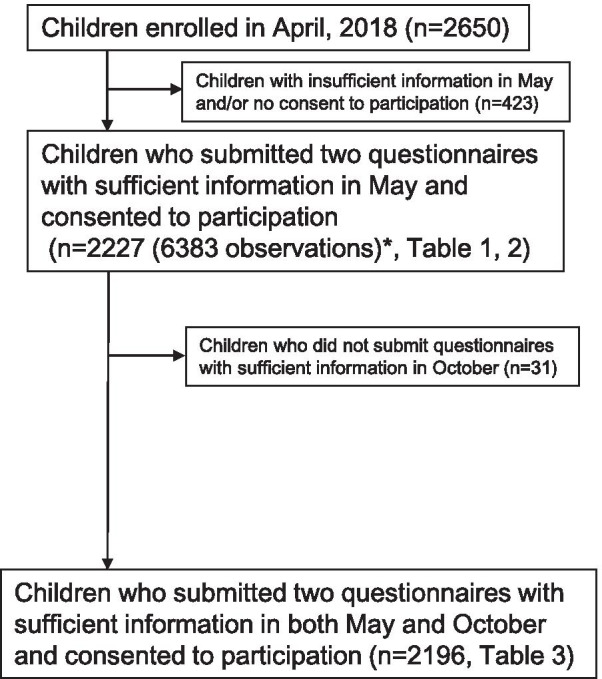
Flow diagram of participant selection for analysis. *In this study, the participants submitted a set of the questionnaires three times (May, October, and February). ‘Observation’ number indicates the number of analyzed questionnaires: if all questionnaires had been submitted, ‘observation’ would have been equal to triple the number of participants. However, as some questionnaires were missing in October and February, ‘observation’ is less than triple

Among 2650 children in the collaborating schools, 2227 children who submitted two questionnaires in May with sufficient information (percentage of correct answers in the nutrition knowledge questionnaire, energy intake estimated by BDHQ15y between 600 kcal and 4500 kcal, and no missing answers for questions using the statistical models) and consent to participate were included in the analysis (Fig. 2). Regarding the guardians, those providing information about the relationship with their children and a nutrition knowledge score were selected from among all guardians of the 2227 analyzed children. Finally, 2085 guardians were included in the analysis. Power calculation was performed using nQuery advanced (Statsols, Cork, Ireland). In a hierarchical 2-level mixed effects model with 7 and 7 clusters in the First and Second groups, respectively, and with 150 subjects per cluster (i.e. 1050 subjects in each group), 88.52% power is achieved to detect a difference of at least 8 (nutrition knowledge score (%)), assuming that the standard deviation between subjects is 12 and that the test is performed at the 5% significance level.

**Fig. 3:**
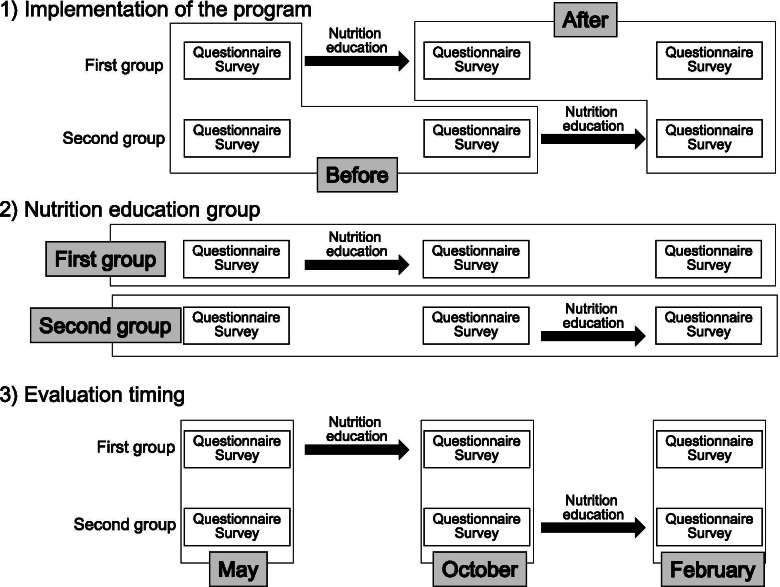
Definition of variables in linear mixed models. Differences in the definitions of three variables (Implementation of the program, Nutrition education group, Evaluation timing) are shown. “Implementation of the program (before vs after the program)” was a major independent variable in showing the effect of the nutrition education program. “Nutrition education group (First vs Second group)” was a covariate used to adjust the difference in knowledge at baseline between the two groups. “Evaluation timing (May vs October or February)” was also a covariate used to adjust the difference in knowledge in the time course

Characteristics of the participants were compared between the First group and the Second group using the t test for continuous variables and the chi-square test for categorical variables. Factors associated with the children’s nutrition knowledge were then assessed by linear mixed models. We used all data obtained in the three surveys (May, October, and February). Consequently, 6383 observations were included for 2227 children. In this analysis, nutrition knowledge level at all survey timings was a dependent variable and implementation of the nutrition education program (before vs after the program; Fig. 3) was a major independent variable. The survey area and participant’s identification code were included as random effects in the models to consider the clustering of participants within the areas and the repetition of the surveys. Covariates included as fixed effects in Model 1 were sex, grade, nutrition education group (First vs Second group, ie. the difference in knowledge at baseline between the two groups (Fig. 3)), and evaluation timing (May vs October or February (Fig. 3)). In Model 2, answers to the four questions about attitude/behavior toward diet were included. Additionally, Model 3 included information collected from the guardians, namely subjective SES, guardians’ nutrition knowledge level, and guardians’ answers to six questions about attitude/behavior toward diet. The number of observations analyzed in Model 3 was 5352 due to missing information for some guardians. The categories of SES were reclassified into the three levels of “poor,” which included those who answered very straitened and straitened; “average,” including those answered average; and “affluent,” including those answered affluent or very affluent.

Further, we investigated factors associated with change in nutrition knowledge (differences between nutrition knowledge in October versus May) in the children by linear mixed models. In this analysis we analyzed 2196 children due to non-return of follow-up questionnaires in October (Fig. 2). The models included change in nutrition knowledge as a dependent variable and nutrition education group (First vs Second group) as a major independent variable. Survey area was included as a random effect. Covariates included in Model 1C (C = change) were sex, grade, and children’s nutrition knowledge level in May (baseline). Model 2C included the children’s answers to the same four questions in Model 2. Model 3C for also included the same variables as in Model 3 and change in guardians’ nutrition knowledge level.

As a supplementary analysis, we summarized changes in food intake in the participants who had sufficient information about food intake and characteristics in all three surveys (May, October, and February). Sex- and grade (children)/age (guardians)-adjusted food intakes were calculated using analysis of covariance by groups (First vs. Second). Change in intake between May and October was calculated, and compared between the First (intervention) and Second (control) group. The same mixed model with Model 1C described above was used for this comparison; namely, the model included food intake (g/1000 kcal) as a dependent variable and implementation of nutrition education as an independent variable. The survey area was included as a random effect in the model, and sex, grade (children)/age (guardians), and corresponding food intake at baseline (children/guardians) were included as fixed effects.

All analyses were performed with Statistical Analysis System (SAS) version 9.4 software (SAS Institute, Cary, NC, USA). Statistical tests were two-sided, and *P* values of < 0.05 were considered statistically significant.

## Results

Characteristics of the participants are summarized in Table 1. All schools completed the nutrition education program on schedule. The percentage of children who thought being careful about what they eat was beneficial and who discussed foods and nutrition with their guardians increased with time. In particular, the proportion of children who discussed diet with their guardians increased from 37.3% in May to 47.5% in October in the First group (i.e. intervention group), but increase of the proportion was small (42.9% in May and 43.3% in October) in the Second group (i.e. control group). In contrast, guardian attitudes did not clearly change, but the percentage who discussed foods and nutrition with their children slightly increased, from 68.2% in May to 74.3% in February among all analyzed guardians. Similarly to the children, this increase was larger after the intervention (67.9% in May and 75.1% in October in the First group; 68.6% in May and 70.0% in October in the Second group).

**Table 1:** Characteristics of Participants (children: *n* = 2227 in May; guardians: *n* = 2085 in May)

	Variable		n (%) or mean, SD		First group (*n* = 1088)^a^		Second group (*n* = 1139)^a^		*P* value^b^
			Total (n = 2227)						
Children	Sex	Boy	1091	(49.0)	543	(49.9)	548	(48.1)	0.40
		Girl	1136	(51.0)	545	(50.1)	591	(51.9)	
	Grade	5th Grader (10–11 y/o)	1115	(50.1)	554	(50.9)	561	(49.3)	0.43
		6th Grader (11–12 y/o)	1112	(49.9)	534	(49.1)	578	(50.8)	
	NK score (%)^c^	mean, SD May *n *= 2227	69.7,	13.1	70.7,	13.8	68.7,	12.3	< 0.001
		October *n* = 2196	75.2,	13.8	80.7,	12.8	70.0,	12.7	< 0.001
		February *n* = 2147	78.4,	12.2	78.9,	12.2	77.9,	12.3	0.054
		Difference b/w O and M *n* = 2196	5.5,	13.4	9.9,	14.1	1.3,	11.1	< 0.001
		Difference b/w F and O *n* = 2128	3.1,	12.2	−1.7,	11.5	7.8,	10.9	< 0.001
Being careful about what you eat is beneficial for you^d^									
		Yes in May *n* = 2227	1782	(80.0)	868	(79.8)	914	(80.3)	0.78
		Yes in October *n* = 2189	1875	(85.7)	928	(87.0)	947	(84.4)	0.09
		Yes in February *n* = 2138	1906	(89.2)	937	(89.3)	969	(89.0)	0.80
Being careful about what you eat earns you esteem from those around you^d^									
		Yes in May *n* = 2227	713	(32.0)	341	(31.3)	372	(32.7)	0.51
		Yes in October *n* = 2189	651	(29.7)	329	(30.8)	322	(28.7)	0.27
		Yes in February *n* = 2138	713	(33.4)	319	(30.4)	394	(36.2)	0.005
Being careful about what you eat places a burden on you^d^									
		Yes in May *n* = 2227	550	(24.7)	282	(25.9)	268	(23.5)	0.19
		Yes in October *n* = 2188	555	(25.4)	270	(25.3)	285	(25.4)	0.95
		Yes in February *n* = 2138	539	(25.2)	275	(26.2)	264	(24.2)	0.29
You discuss meals, food, nutrition, etc. with your guardians^d^									
		Yes in May *n* = 2227	895	(40.2)	406	(37.3)	489	(42.9)	0.007
		Yes in October *n* = 2192	994	(45.4)	507	(47.5)	487	(43.3)	0.051
		Yes in February *n* = 2138	1055	(49.4)	505	(48.1)	550	(50.5)	0.27
Guardians			Total (n = 2085)e		First group (*n* = 1023)e		Second group (*n* = 1062)e		
	Relationship	Mother	1910	(91.6)	936	(91.5)	974	(91.7)	0.95
		Father	122	(5.9)	59	(5.8)	63	(5.9)	
		Grandmother	13	(0.6)	7	(0.7)	6	(0.6)	
		Other	40	(1.9)	21	(2.1)	19	(1.8)	
	Age (y/o)	mean, SD	41.5,	5.5	42.0,	5.5	41.1,	5.4	< 0.001
	Subjective SES	Poor	645	(31.4)	301	(29.9)	344	(32.8)	0.049
		Average	1157	(56.3)	564	(56.1)	593	(56.5)	
		Affluent	253	(12.3)	141	(14.0)	112	(10.7)	
	NK score (%)^c^	mean, SD May *n* = 2085	68.2,	15.4	69.6,	14.9	66.8,	15.8	< 0.001
		October *n* = 1892	70.2,	14.7	71.6,	14.3	68.8,	15.0	< 0.001
		February *n* = 1768	71.6,	14.0	72.9,	13.6	70.4,	14.3	< 0.001
		Difference b/w O and M *n* = 1792	1.8,	10.4	1.9,	10.1	1.8,	10.6	0.72
		Difference b/w F and O *n* = 1611	1.0,	9.7	0.7,	9.7	1.3,	9.6	0.24
Being careful about what you eat is beneficial for you^d^									
		Yes in May *n* = 2066	1943	(94.1)	957	(94.2)	986	(93.9)	0.78
		Yes in October *n* = 1865	1742	(93.4)	858	(94.7)	884	(92.2)	0.03
		Yes in February *n* = 1754	1667	(95.0)	811	(95.1)	856	(95.0)	0.95
Being careful about what you eat earns you esteem from those around you^d^									
		Yes in May *n* = 2059	871	(42.3)	435	(42.9)	436	(41.7)	0.56
		Yes in October *n* = 1864	772	(41.4)	380	(42.0)	392	(40.9)	0.63
		Yes in February *n* = 1748	798	(45.7)	401	(47.1)	397	(44.3)	0.23
Being careful about what you eat places a burden on you^d^									
		Yes in May *n* = 2062	875	(42.4)	429	(42.3)	446	(42.6)	0.91
		Yes in October *n* = 1866	758	(40.6)	361	(39.9)	397	(41.4)	0.51
		Yes in February *n* = 1753	713	(40.7)	351	(41.2)	362	(40.2)	0.69
You discuss meals, food, nutrition, etc. with your child^d^									
		Yes in May *n* = 2052	1400	(68.2)	682	(67.9)	718	(68.6)	0.73
		Yes in October *n* = 1868	1353	(72.4)	680	(75.1)	673	(70.0)	0.01
		Yes in February *n* = 1754	1303	(74.3)	639	(74.9)	664	(73.7)	0.56
My current dietary habits are firmly fixed, so changing them is difficult^d^									
		Yes in May *n* = 2064	814	(39.4)	375	(37.0)	439	(41.9)	0.02
My dietary habits are healthy, so there’s no need to change them^d^									
		Yes in May *n* = 2063	393	(19.1)	189	(18.6)	204	(19.5)	0.63

*b/w* Between, *F* February, *M* May, *NK score* Nutrition knowledge score, *O* October, *SES* Socioeconomic status, *y/o* Years old

^a^ First group means the participant group which received the nutrition education program from June to September. Second group means those who received it from November to January

^b^ Comparison of characteristics between the First and Second groups was performed using the t test for continuous variables and χ2 test for categorical variables

^c^ NK score is the percentage (%) of correct answers (regarded as nutrition knowledge level) in the nutrition knowledge questionnaires

^d^ Percentage who answered ‘Yes’ for the question was calculated. As shown, the total number of responders (i.e. denominator of the calculation) differed by timing of the survey (May, October, and February)

^e^ The numbers shown here includes guardians of 2227 children with information about the relationship with their children (mother, father, grandmother, or others) and NK score

Factors associated with children’s nutrition knowledge are shown in Table 2. According to Model 3, nutrition knowledge after the nutrition education program was 8.7% (95%CI, 7.7–9.7) higher than before. Among children who thought that being careful about what he/she eats was beneficial, the nutrition knowledge level was 4.1% (95%CI, 3.3–5.0) higher than among children who did not think so. Further, when a guardian thought in the same way, the knowledge level of his/her child was 1.8% (95%CI, 0.57–3.1) higher than a child whose guardian did not think so. Discussion about food/nutrition between children and guardians was significantly related with higher children’s nutrition knowledge. When a child answered that he/she discussed meals, food, nutrition, etc. with his/her guardians, the nutrition knowledge level was 1.3% (95%CI, 0.72–1.9) higher than for a child who answered that he/she did not discuss these with his/her guardians. Subjective lower SES was also significantly associated with the children’s lower nutrition knowledge. When a guardian thought that his/her SES was lower than the average, the knowledge level of his/her child was 1.3% (estimates − 1.3, 95%CI: − 2.2, − 0.30) lower than for a child whose guardian thought that his/her SES was the average. Guardians’ higher nutrition knowledge was significantly associated with children’s higher nutrition knowledge. When a guardian’s nutrition knowledge level was 1.0% higher, his/her child’s nutrition knowledge was 0.13% (95%CI, 0.10–0.15) higher.

**Table 2:** Factors Associated with Children’s Nutrition Knowledge by Linear Mixed Models^a^ (*n* = 2227, obs = 6383)

R	Variable or question (Fixed Effects)		Model 1		Model 2		Model 3 (obs = 5352)^b^	
			Estimates (% of correct answers)	95%CI	Estimates (% of correct answers)	95%CI	Estimates (% of correct answers)	95%CI
	Intercept		68.0	[65.9, 70.1]	64.4	[62.4, 66.4]	54.3	[51.6, 57.0]
C	Sex	Girl (vs Boy)	1.1	[0.24, 1.9]*	0.79	[−0.04, 1.6]	0.30	[− 0.54, 1.1]
C	Grade	6th Grader (vs 5th)	3.4	[2.6, 4.3]*	3.3	[2.5, 4.1]*	3.4	[2.5, 4.2]*
	Nutrition education program: group^c^	Second group (vs First)	−1.5	[−2.4, − 0.62]*	− 1.6	[− 2.5, − 0.74]*	− 1.4	[− 2.3, − 0.48]*
	Nutrition education program: implementation^c^	After (vs Before)	9.0	[8.1, 9.9]*	8.7	[7.8, 9.6]*	8.7	[7.7, 9.7]*
	Nutrition education program: evaluation timing^c^	October (vs May)	1.2	[0.51, 1.9]*	1.03	[0.36, 1.7]*	1.3	[0.52, 2.0]*
		February (vs May)	0.01	[−1.0, 1.0]	−0.21	[− 1.3, 0.82]	− 0.32	[− 1.5, 0.82]
C	Being careful about what you eat is beneficial for you.^d^	Yes (vs No)			4.2	[3.4, 4.9]*	4.1	[3.3, 5.0]*
C	Being careful about what you eat earns you esteem from those around you.^d^	Yes (vs No)			0.71	[0.10, 1.3]*	0.70	[0.04, 1.4]*
C	Being careful about what you eat places a burden on you.^d^	Yes (vs No)			−0.76	[−1.4, − 0.13]*	− 0.68	[−1.4, 0.01]
C	You discuss meals, food, nutrition, etc. with your guardians.^d^	Yes (vs No)			1.3	[0.72, 1.9]*	1.3	[0.68, 1.9]*
G	Subjective SES	Poor (vs Average)					−1.3	[−2.2, −0.30]*
		Affluent (vs Average)					−0.66	[−2.0, 0.68]
G	Guardians’ NK level	in May					0.13	[0.10, 0.15]*
G	Being careful about what you eat is beneficial for you.^d^	Yes (vs No)					1.8	[0.57, 3.1]*
G	Being careful about what you eat earns you esteem from those around you.^d^	Yes (vs No)					0.14	[−0.49, 0.77]
G	Being careful about what you eat places a burden on you.^d^	Yes (vs No)					−0.42	[−1.1, 0.23]
G	You discuss meals, food, nutrition, etc. with your child.^d^	Yes (vs No)					0.62	[−0.09, 1.3]
G	My current dietary habits are firmly fixed, so changing them is difficult.^e^	Yes in May (vs No)					−0.65	[−1.5, 0.23]
G	My dietary habits are healthy, so there’s no need to change them.^e^	Yes in May (vs No)					1.3	[0.16, 2.4]*

C, child; G, guardian; NK, nutrition knowledge; obs, observation; R, respondent; SES socioeconomic status; *P < 0.05

^a^ Number of participants included in the analysis was 2227, and number of included observations were 6383, because most participants answered the questionnaire three times (May, October, and February). Mixed models included nutrition knowledge score (the percentage (%) of correct answers in the nutrition knowledge questionnaire) as a dependent variable and implementation of nutrition education as an independent variable. Other variables in the table were covariates. Participants’ ID number and survey area were included as random effects in the models, and other covariates were treated as fixed effects

^b^ Due to missing guardian answers, number of observations used in analysis by Model 3 was smaller than those in Models 1 and 2

^c^ Effect of nutrition education groups were assessed after separating three parts: Group itself (First vs Second group), Implementation of the nutrition education (before vs after implementation), and Evaluation timing (May, October, and February)

^d^ These questions were asked three times (May, October, and February), and each answer was used for the corresponding observation

^e^ These questions were asked only in May. For observations in October and February, answers in May were extrapolated

Factors associated with change in children’s nutrition knowledge from May to October are shown in Table 3. By the end of October, children in the First group had finished the nutrition education program whereas those in the Second group had not. Mean change in nutrition knowledge level was 9.8% (95%CI, 8.8–10.8) higher in the First group. Nutrition knowledge level in May was negatively associated with the change in knowledge (− 0.54% (95%CI, − 0.58 to − 0.50)). In other words, the increase in nutrition knowledge was significantly smaller in the children whose nutrition knowledge was higher at baseline. Higher guardian nutrition knowledge at baseline and a larger increase in guardian nutrition knowledge were significantly associated with a larger increase in children’s knowledge. When a guardian’s nutrition knowledge was 1% higher at baseline, the increase in nutrition knowledge in his/her child was 0.11% (95%CI, 0.07–0.16) larger. When the guardians answered that they discussed diet with their children, the increase in children’s nutrition knowledge was significantly larger, at 1.5% (95%CI, 0.43-2.7) larger than for a child whose guardian answered that he/she did not discuss this with his/her child.

**Table 3:** Factors Associated with Change in Children’s Nutrition Knowledge from May to October by Linear Mixed Models^a^ (*n* = 2196)

R	Variable or question (Fixed effects)		Model 1C		Model 2C		Model 3C (*n* = 1734)^b^	
			Estimates (% of correct answers)	95%CI	Estimates (% of correct answers)	95%CI	Estimates (% of correct answers)	95%CI
	Intercept		32.6	[29.6, 35.5]	32.2	[29.2, 35.2]	25.9	[21.4, 30.4]
C	Sex	Girl (vs Boy)	1.1	[0.2, 2.0]*	1.0	[0.13, 1.9]*	0.77	[−0.20, 1.7]
C	Grade	6th Grader (vs 5th)	2.6	[1.7, 3.5]*	2.5	[1.6, 3.4]*	2.7	[1.7, 3.7]*
	Nutrition education program: group	First group (vs Second)	9.6	[8.7, 10.5]*	9.6	[8.7, 10.6]*	9.8	[8.8, 10.8]*
C	NK level in May		−0.49	[−0.52, −0.45]*	−0.50	[− 0.54, − 0.46]*	−0.54	[− 0.58, − 0.50]*
C	Being careful about what you eat is beneficial for you.^c^	Yes in May (vs No)			2.2	[1.0, 3.4]*	2.0	[0.68, 3.3]*
C	Being careful about what you eat earns you esteem from those around you.^c^	Yes in May (vs No)			−0.04	[−1.0, 1.0]	−0.14	[− 1.2, 0.95]
C	Being careful about what you eat places a burden on you.^c^	Yes in May (vs No)			−0.52	[−1.6, 0.5]	−0.45	[−1.6, 0.69]
C	You discuss meals, food, nutrition, etc. with your guardians.^c^	Yes in May (vs No)			−0.30	[−1.2, 0.6]	−0.42	[−1.5, 0.61]
G	Subjective SES	Poor (vs Average)					−0.63	[−1.7, 0.48]
		Affluent (vs Average)					−1.4	[−2.9, 0.12]
G	Guardians’ NK level	in May					0.11	[0.07, 0.16]*
G	Change in Guardians’ NK level	May to October					0.06	[0.006, 0.11]*
G	Being careful about what you eat is beneficial for you.^c^	Yes in May (vs No)					1.1	[−1.0, 3.3]
G	Being careful about what you eat earns you esteem from those around you.^c^	Yes in May (vs No)					−0.31	[−1.3, 0.70]
G	Being careful about what you eat places a burden on you.^c^	Yes in May (vs No)					0.33	[−0.70, 1.4]
G	You discuss meals, food, nutrition, etc. with your child.^c^	Yes in May (vs No)					1.5	[0.43, 2.7]*
G	My current dietary habits are firmly fixed, so changing them is difficult.^d^	Yes in May (vs No)					0.24	[−0.78, 1.3]
G	My dietary habits are healthy, so there’s no need to change them.^d^	Yes in May (vs No)					0.56	[−0.71, 1.8]

*C* Child, *G* Guardian, *NK* Nutrition knowledge, *R* Respondent, *SES* Socioeconomic status; *P < 0.05

^a^ Mixed models included change in children’s nutrition knowledge score (%) as a dependent variable and implementation of nutrition education as an independent variable

Other variables in the table were covariates. Survey area was included as a random effect in the models, and other covariates were treated as fixed effects

^b^ Due to missing guardian answers, the number of participants in analysis by Model 3 was smaller than those in Models 1 and 2

^c^ These questions were asked three times (May, October, and February), and answers in May was used in the analysis

^d^ These questions were asked in May only

Food intakes and their change in the children and the guardians are shown in Supplementary Table 1. Change in any food intake from May to October in the children did not significantly differ between the First and Second groups. Regarding the guardians, decrease in vegetable intake was significantly smaller in the First group than in the Second (− 4.8 g/1000 kcal vs. -5.6 g/1000 kcal, *p* = 0.03). Also, increase in soft drink intake was significantly larger in the Second group (− 0.1 g/1000 kcal vs. 2.4 g/1000 kcal, p = 0.03). Increase in fruits intake was marginally larger in the First group (3.2 g/1000 kcal vs. 2.8 g/1000 kcal, *p* = 0.054).

## Discussion

In this study, children’s nutrition knowledge level significantly increased after implementation of the nutrition education program. Although the nutrition knowledge level was higher in the First group than the Second, possibly due to the non-randomized design, the absolute effect of the intervention was still significant after adjusting for the difference between groups by the linear mixed models. In addition, discussion between the children and their guardians about diet was associated with higher nutrition knowledge in the children. The nutrition education in this study increased the proportion of children and guardians who discussed diet with their guardians/children, and this point is a strength of the program. The findings that the children’s and guardians’ knowledge levels were significantly associated, and that the increase in children’s knowledge was significantly larger when their guardians’ knowledge and its increase was larger, indicate that nutrition education for both parties and communication between them is a key to nutrition literacy acquisition.

In addition to the facilitation of communication, the proportion of the children who considered “being careful about what you eat is beneficial” increased after the intervention. Agreement with this statement was significantly associated with higher nutrition knowledge in the children, as well as with a larger increase in the children’s knowledge after the intervention. Based on the Knowledge-Attitude-Behavior (KAB) model [17], we may have observed an attitude change after accumulation of knowledge. This could have resulted in behavioral change; that is, the observed change might lead to better dietary intake among the children in the future. Previous research has shown a relationship between a positive attitude towards healthy eating behavior and healthy dietary intake, such as with a higher intake of vegetables and fruits [18]. However, the KAB model assumes that people are rational [17], and is operative only in limited subsets of motivated people [19]. In the present study, we assessed food intake in the children and their guardians (Supplementary Table 1). Unfortunately, food intakes were not apparently changed by the intervention in the children. This finding may be plausible, given that the menus of school lunches were not changed and the children consumed foods provided by their guardians. Establishing a role for guardians in a dietary intervention therefore appears to be important even when the main target of the intervention is children. On the other hand, the change in food intake in the guardians seemed slightly better in the First (intervention) group than the Second (control) group. Since the increase in nutrition knowledge was small in the guardians, continuity of education, intensive inclusion of the guardians into the nutrition education program, and environmental improvement (e.g. easy access to fruits and vegetables) might be important as a means of achieving better dietary intakes in both the children and the guardians. The guardians’ vegetable intake decreased in both groups, which may have been due to seasonal change in food choices.

Another important finding of this study was that a lower nutrition knowledge level at baseline was significantly associated with a larger increase in knowledge in the children. Although this result might have been affected by ‘regression to the mean’, children’s nutrition knowledge level in the lowest quartile increased substantially, from 53.0% in May to 70.7% in February (data not shown in the tables). In contrast, that of the highest quartile slightly decreased during this period, from 85.9 to 84.3%. Since a school-based nutrition education program can provide a uniform intervention to all children, implementation may result in a decrease in knowledge disparity. Public schools may be good places to implement population approaches without increasing inequality for children [7]. SES was significantly associated with the nutrition knowledge level of the children, but the change in nutrition knowledge by the education program was the same between those with average SES and poor SES. This result also supports the potential of school-based nutrition education to reduce the knowledge disparity. Similar results (lower educational status was associated with larger increase in nutrition knowledge score following nutrition education) were also observed in a study in pregnant women [20].

To date, several reports of school-based nutrition education for upper graders in primary schools have been reported. Lakshman et al. reported an intervention study based on a program which included a card game and a package of classroom activities [21]. Results showed a modest increase in nutrition scores, but 10 of the 38 recruited schools failed to complete the intervention due to time pressure from the school curriculum. Scherr et al. reported another intervention study which included classroom education and an approach to the family [22]. They distributed newsletters to the families and held health fairs, but the paper does not mention communication between the children and families. Finally, Carraway-Stage et al. reported an interesting attempt to integrate nutrition and science education [23]; results showed that their program efficiently conveyed knowledge about nutrition, and improved nutrition knowledge.

Our study has several strengths. The large number of subjects and high response rate (84.0% in children and 78.7% in guardians) supported the high power of the statistical analysis and low selection bias. Nutrition knowledge was evaluated using validated questionnaires in both the children and the guardians [10], and the effect of the guardians’ factors on the children’s nutrition knowledge was investigated in addition to the effect of the children’s factors. Communication between the children and the guardians was also examined. Linear mixed models were used to consider repeated measurements and multilevel structure of variables. The statistical models included possible confounding factors such as sex, grade, SES, and attitude toward diet.

Several limitations of this study also warrant mention. First, since all the primary schools were in the same prefecture, the participants may not have represented the general Japanese population. However, the prefecture is located in the central part of the main island of Japan and includes both urban and rural areas, and the survey areas covered all five administrative districts of the prefecture Second, the intervention was not assigned randomly. We selected two schools with similar characteristics from each survey area, and assigned the intervention by area. Also, mixed models were used to adjust for differences in the two groups themselves, apart from the effect of intervention. Finally, since most information was collected by self-administered questionnaire, objectivity could have been impaired for some variables. For example, SES was self-reported and household income was not investigated, due to the difficulty in collecting personal information at public schools.

## Conclusions

This nutrition education program, which included a classroom lecture and homework, increased the nutrition knowledge of primary school children. The homework was aimed at facilitating communication between the children and their guardians, and fulfilled this function well. The present study also shows that public schools can be crucial venues for decreasing disparities in nutrition knowledge. If efficient health education programs, including nutrition education, are provided for all children, future health disparities can be reduced.

## Supplementary Information

**Additional file 1: Supplementary Table 1** Food intakes and their change from May to October among children (*n* = 1998) and guardians (*n* = 1300).

## Data Availability

The datasets analyzed during the current study are available from the corresponding author on reasonable request.

## Abbreviations

BDHQ: Brief-type self-administered diet history questionnaire
CI: Confidence interval
KAB model: Knowledge-Attitude-Behavior model
SES: Socioeconomic status
