# Supplementary material for: A school-based nutrition education program involving children and their guardians in Japan: facilitation of guardian-child communication and reduction of nutrition knowledge disparity
Source: Nutr J. 2021 Nov 18;20:92. doi: 10.1186/s12937-021-00751-z (PMC8600693; doi:10.1186/s12937-021-00751-z)
Supplement: Supplementary file 1 — Additional file 1: Supplementary Table 1 Food intakes and their change from May to October among children (n = 1998) and guardians (n = 1300). [file 12937_2021_751_MOESM1_ESM.pdf]

Supplementary Table 1. Food intakes and their change from May to October among children (n=1998) and guardians (n=1300)[a]

		First group (intervention group in October)[b]				Second group (control group in October)[b]				p value[e] (Comparison b/w X and Y)
		Survey timing			Change b/w Oct and May[d] (X)	Survey timing			Change b/w Oct and May[d] (Y)	
		May	October	February		May	October	February		
Child	Number of respondents	978	978	978		1020	1020	1020		
	Nutrition knowledge (%)	70.9	80.8	79.4	9.9	69.1	70.5	78.3	1.3	<0.001
	Food intake (adjusted mean, g/1000kcal)[c]									
	Staple food[e]	223	237	244	13.3	225	234	243	9.1	0.18
	Soybeans and soy products	22	21	23	-1.0	21	21	23	-0.04	0.74
	Vegetables[f]	99	96	110	-2.8	87	87	96	0.1	0.15
	Fruits	29	29	36	0.5	24	25	33	1.7	0.25
	Fish	28	28	28	0.3	29	29	28	-0.04	0.92
	Meat	37	36	35	-0.5	36	36	35	0.001	0.77
	Milk and dairy products	114	115	108	1.3	111	112	110	1.1	0.85
	Sweets and snacks	47	45	39	-1.6	49	47	40	-2.3	0.67
	Soft drinks[g]	64	61	54	-3.3	70	68	63	-2.1	0.30
Guardian	Number of respondents	636	636	636		664	664	664		
	Nutrition knowledge (%)	71.3	73.0	74.4	2.6	69.1	70.1	71.8	1.8	<0.001
	Food intake (adjusted mean, g/1000kcal)[c]									
	Staple food[f]	224	224	228	-0.03	235	225	232	-10.2	0.07
	Soybeans and soy products	34	33	35	-1.1	30	32	34	1.1	0.56
	Vegetables[g]	130	126	134	-4.8	117	112	124	-5.6	0.03
	Fruits	26	29	32	3.2	21	24	30	2.8	0.054
	Fish	36	39	38	3.2	33	38	37	5.4	0.35
	Meat	45	45	46	-0.2	44	45	45	1.2	0.29
	Milk and dairy products	56	57	62	1.1	52	56	59	4.2	0.60
	Sweets and snacks	38	34	32	-3.5	38	35	33	-3.0	0.61
	Soft drinks[h]	27	27	17	-0.1	31	33	21	2.4	0.03

b/w, between; Oct, October

[a] Participants included in Table 1 having sufficient information about food intakes and characteristics the in three surveys (May, October, and February) were included in the analysis.

[b] Nutrition education was given between June and September for the First group and between October and January for the Second group.
Thus, in October, the First group was an intervention group (after the intervention) and the Second group was a control group (before the intervention).

[c] Food intakes adjusted for sex and grade/age by analysis of covariance are shown.

[d] Differences were calculated as follows: food intakes in October - those in May. Those adjusted for sex and grade/age by analysis of covariance are shown.

[e] Comparison of changes between the First group and the Second group are shown. The p values for food intakes were calculated by a mixed model which included the change in a food intake (g/1000kcal) as a dependent variable and implementation of nutrition education as an independent variable.
Survey area was included as a random effect in the model, and sex, grade (children)/age(guardians), and corresponding food intake at baseline (children or guardians) were included as fixed effects.

[f] Intake of staple foods was the sum of rice, bread, and noodle intake.

[g] Vegetables includes seaweeds and mushrooms.

[h] Soft drinks includes soda, sugar-sweetened juice, coffee with milk, and fermented lactic beverages.
